# Scalable, highly stable Si-based metal-insulator-semiconductor photoanodes for water oxidation fabricated using thin-film reactions and electrodeposition

**DOI:** 10.1038/s41467-021-24229-y

**Published:** 2021-06-25

**Authors:** Soonil Lee, Li Ji, Alex C. De Palma, Edward T. Yu

**Affiliations:** 1 Microelectronics Research Center, University of Texas, Austin, TX USA; 2 State Key Laboratory of ASIC and System, School of Microelectronics, Fudan University, Shanghai, China

**Keywords:** Photocatalysis, Devices for energy harvesting, Electrical and electronic engineering, Materials for energy and catalysis

## Abstract

Metal-insulator-semiconductor (MIS) structures are widely used in Si-based solar water-splitting photoelectrodes to protect the Si layer from corrosion. Typically, there is a tradeoff between efficiency and stability when optimizing insulator thickness. Moreover, lithographic patterning is often required for fabricating MIS photoelectrodes. In this study, we demonstrate improved Si-based MIS photoanodes with thick insulating layers fabricated using thin-film reactions to create localized conduction paths through the insulator and electrodeposition to form metal catalyst islands. These fabrication approaches are low-cost and highly scalable, and yield MIS photoanodes with low onset potential, high saturation current density, and excellent stability. By combining this approach with a p^+^n-Si buried junction, further improved oxygen evolution reaction (OER) performance is achieved with an onset potential of 0.7 V versus reversible hydrogen electrode (RHE) and saturation current density of 32 mA/cm^2^ under simulated AM1.5G illumination. Moreover, in stability testing in 1 M KOH aqueous solution, a constant photocurrent density of ~22 mA/cm^2^ is maintained at 1.3 V versus RHE for 7 days.

## Abstract (web summary)

Authors demonstrate Si-based MIS photoanodes using Al thin-film reactions to create localized conduction paths through the insulator and Ni electrodeposition to form metal catalyst islands. These approaches yielded low onset potential, high saturation current density, and excellent stability.

## Introduction

Photoelectrochemical (PEC) water splitting is a promising technology for converting solar energy into clean and storable chemical energy. In PEC cells, semiconductors play a key role in absorbing photons from the light source to create mobile charge carriers. Various semiconductor materials have been studied for the high-performance PEC cells, including metal oxides^1–4^, nitrides^5,6^, Si^7–17^, III–V compound semiconductor materials^18–20^, and others^21,22^. Among these, Si-based photoelectrodes have attracted substantial interest due to silicon’s moderate bandgap (1.12 eV), high charge mobility and diffusion lengths, and well-established technological infrastructure^23^. However, Si-based photoanodes for the oxygen evolution reaction (OER) remain challenging to engineer due to the complex four-electron reaction mechanism which requires a large overpotential, and poor chemical stability in alkaline solutions. To improve the OER performance of Si-based photoanodes, metal-insulator-semiconductor (MIS) structures have been widely used for Si-based photoanodes due to their high efficiency and improved stability^9–11,14–16^. An efficient metal catalyst at the surface improves the reaction kinetics of photoanodes, reducing the overpotential for OER^17,24–26^. Corrosion of Si in aqueous electrolytes is suppressed in MIS photoelectrode structures by protecting the Si surface using thin layers of insulators such as TiO_2_^13,27,28^, NiO_*X*_^9,11,12^, SrTiO_3_^8^, SiN_*X*_^29^, and SiO_*X*_^9–11,14–16^.

**Fig. 1:**
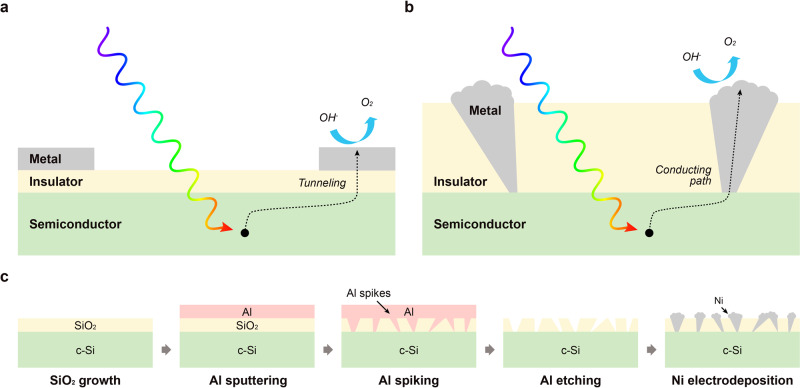
Schematics of metal-insulator-semiconductor photoanodes. **a** Schematic illustration of conventional approach for photogenerated carrier transport, via tunneling, across thin electrically insulating layer in MIS photoelectrode. **b** Schematic illustration of localized metallic conduction paths through a thick electrically insulating layer, enabling the use of much thick insulators that provide high stability in an MIS photoelectrode. **c** Schematic illustration of a highly scalable, nonlithographic fabrication process for realizing structure shown in **b**.

For a Si-based MIS photoanode, minority carriers are generated by illumination of the semiconductor and, typically, extracted to the metal layer by tunneling through the insulator, as shown in Fig. 1a. The tunneling current density decreases exponentially with increasing insulator layer thickness so that, with few exceptions^10^, ultrathin insulators under 5 nm are needed for efficient MIS photoanodes^30^. However, it has also been reported that MIS photoelectrodes with thin protective insulator layers can be susceptible to corrosion of the semiconductor in alkaline solutions, so that insulator layer thicknesses above 50 nm are required for long-term stability in alkaline solutions^31,32^. Due to this tradeoff between efficiency and stability, optimizing the insulating layer thickness is key to the OER performance of MIS photoanodes.

Our previous work demonstrated a new approach to increasing the effective conductivity of a thick insulating layer in MIS photoelectrodes by electrically inducing the localized dielectric breakdown of the insulating region beneath metal catalyst islands^10^. Since the breakdown process resulted in the formation of localized conductive paths through the thick oxide layer, photogenerated minority carriers are easily transported via the conductive path instead of by tunneling. Using this approach, improved photocurrents were observed with good long-term stability due to the thick insulating layers. However, this method requires complex and time-consuming processing techniques for the formation of localized breakdown paths, along with lithographic patterning of the top metal layer. Recently, Loget et al.^11^ and Zhao et al.^12^ reported on the deposition of Ni catalysts for the metal layer of MIS photoanodes using electrodeposition and electroless deposition, respectively. These methods yielded well-defined Ni deposition for the MIS photoanode and enabled efficient OER performance without using complex lithographic techniques. However, stability in high pH aqueous solutions was limited to 40 h or considerably less, potentially due to the absence of an insulating protective layer of sufficient quality and thickness.

Herein, we demonstrate a low-cost and highly scalable method for producing high-performance and very stable Si-based MIS photoanodes by exploiting a thin-film reaction of Al through an insulating oxide layer followed by Ni electrodeposition, and not requiring any lithographic patterning. The thin-film reaction of Al with SiO_2_ or Si, leading to localized penetration of Al “spikes” into the underlying material, has been studied extensively since it can cause an electrical short in silicon pn-junction structures with Al contact metallization. Al spiking can also occur through an insulating SiO_2_ layer, and has been exploited to form Ohmic contacts through an oxide passivation layer on Si^33,34^. In an MIS photoelectrode structure with Al as the metal layer, annealing of the Al/SiO_2_/Si structure above 300 °C causes Al to penetrate the underlying SiO_2_ and induces the formation of localized metal spikes in the MIS structure. As shown in Fig. 1b, each metal spike provides a local conductive path through the thick SiO_2_ layer. Typical spike densities are ~10^8^–10^9^ cm^−2^, enabling a very efficient collection of photogenerated carriers. The surrounding regions of oxide remain electrically insulating and retain their protective functionality. In this work, after the formation of localized Al spikes through the SiO_2_ layer, the Al is etched and replaced, via electrodeposition, by Ni, which serves as the OER catalyst. During the electrodeposition process, Ni covers the exposed Si surface resulting in the growth of dispersed Ni nano-islands on the SiO_2_ surface at the corresponding locations. The remaining exposed thick SiO_2_ and the electrodeposited Ni have excellent corrosion resistance in alkaline aqueous solutions. This process results in the formation of Si-based MIS photoanodes with high efficiency, good long-term stability, low cost, and high manufacturability without using any complex and costly lithographic patterning techniques.

## Results and discussion

### Al thin-film reaction on Si-based MIS photoanode

Figure 1c shows the fabrication process for a Si MIS photoanode using the Al/SiO_2_ thin-film reaction to form localized Al spikes. As shown schematically in Fig. 1b, to help ensure long-term stability of the photoanode, 90 nm thick SiO_2_ layers were formed by thermal oxidation to serve as the insulator of the MIS structure^31^. A 90 nm thick SiO_2_ layer also provides low optical surface reflectance in water in the 400–900 nm wavelength range (Supplementary Fig. 1). To form localized Al paths through the SiO_2_ insulating layer, 100 nm Al was deposited by DC sputtering, followed by annealing. During the annealing process, the Al penetrates locally through the SiO_2_ layer and contacts the Si substrate. Since this Al spiking thin-film reaction occurs in localized areas across the whole surface, an array of localized contacts between the metal and semiconductor layers is formed within the Al/SiO_2_/Si structure. Since the Al film is easily corroded in solution, however, it is not suitable for the metal layer of an MIS photoanode. Therefore, the Al was replaced by Ni, which shows good stability and acts as an OER catalyst. The Al layer was first etched by Al etchant, leaving exposed the unreacted SiO_2_ surface and the Si surface underlying the areas where the spiking Al reaction occurred. After etching the Al, Ni was deposited on the resulting surface by electrodeposition. During this process, Ni fills the exposed Si areas first, since higher electric fields are present at the exposed Si surface, and then forms Ni nano-islands. As a result, a Ni/SiO_2_/Si MIS photoanode with localized Ni conductive paths through the oxide can be fabricated using the Al thin-film reaction followed by etching and Ni electrodeposition.

**Fig. 2:**
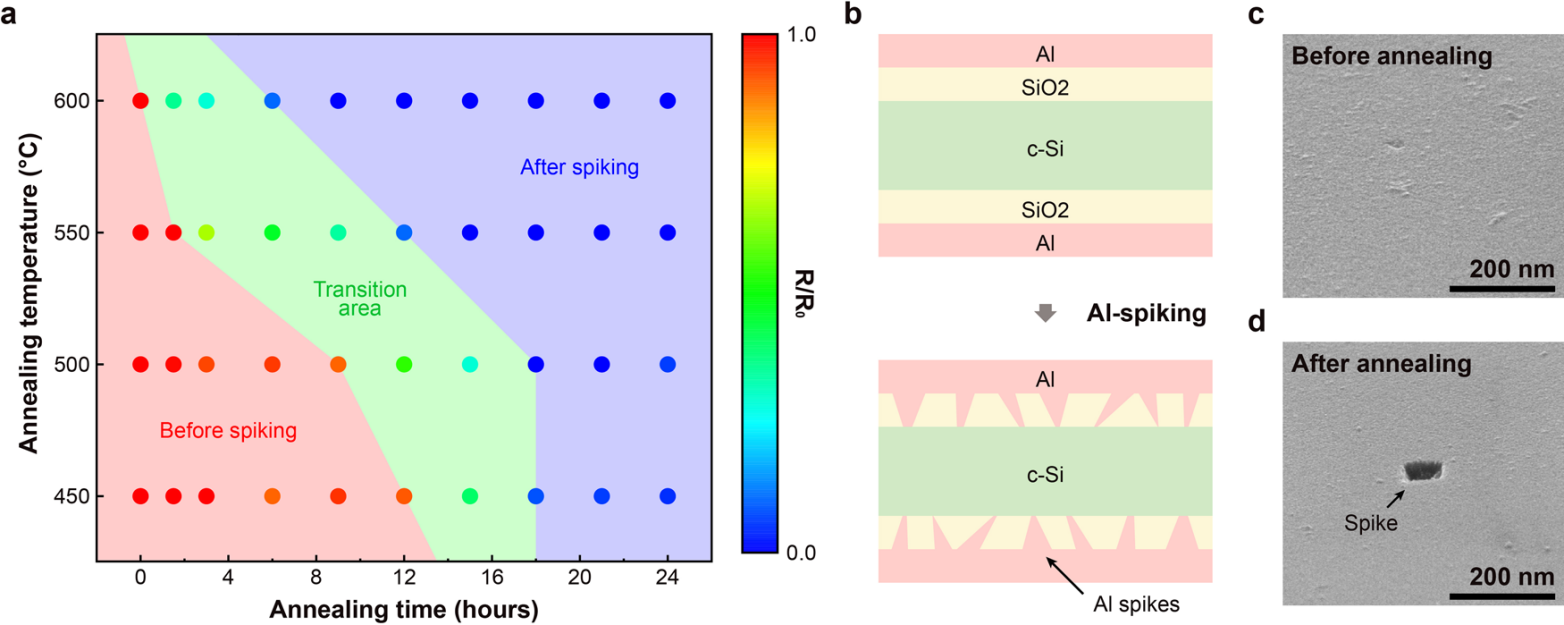
Resistance changes after Al spiking. **a** Electrical resistance of Al/SiO_2_/Si/SiO_2_/Al structure as a function of annealing temperature and duration. **b** Schematic illustration of sample structure evolution upon annealing to induce thin-film reaction between Al and SiO_2_ to form Al “spikes” penetrating the SiO_2_ layers. **c**, **d** Scanning electron micrographs of SiO_2_ surface, before and after annealing. **e** Scanning electron micrographs of SiO_2_ surface before annealing and after 24 h annealing at temperatures of 450–600 °C.

To optimize the Al thin-film reaction and spiking process, a series of Al/SiO_2_/Si/SiO_2_/Al samples were prepared by SiO_2_ growth and Al deposition on both sides of Si substrates, and their series resistance from top to bottom (top-bottom resistance) was measured before and after different annealing processes (Fig. 2a and Supplementary Fig. 2). It has been reported that, in an Al/SiO_2_/Si structure, Al reduces the underlying SiO_2_ layer and penetrates to the Si layer during annealing, the phenomenon referred to as Al spiking^33–35^. The formation of Al spikes requires around 2.56 eV of activation energy for the SiO_2_ reduction process, and can occur at temperature above 300 °C^35^. The top-bottom resistance changes were measured for annealing temperatures of 450–600 °C and durations of 0–24 h as shown in Fig. 2a. Before the annealing process, the top-bottom resistances of the Al/SiO_2_/Si/SiO_2_/Al substrates were relatively high (20–30 Ω cm^2^) due to the thick SiO_2_ insulating layers. In the red-shaded region of Fig. 2a, the high resistance was maintained for the first several hours for all annealing temperatures. However, as the temperature and duration of annealing increased, there was a sharp transition in resistance to ~10% of its initial value (green region in Fig. 2a). After this transition, the resistance reaches ~0.5% of its initial value (blue region in Fig. 2a) for a longer annealing time and/or higher annealing temperature. During the annealing process, Al penetrates locally through the SiO_2_ layers and reaches the underlying Si substrate, forming a metallic path and Ohmic contact between the Al and Si layers as shown in Fig. 2b. The formation of these structures causes an abrupt drop in top-bottom resistance at the transition points. Before the annealing process, the Al layer exhibits uniform morphology on the surface (Fig. 2c). After annealing of an Al/SiO_2_/Si structure at 550 °C for 24 h, localized Al spikes were observed on the surface (Fig. 2d). After etching the Al layer from the Al/SiO_2_/Si substrate, SiO_2_/Si surfaces before and after the annealing process were characterized again using plan-view scanning electron microscopy (SEM). Without any annealing, a uniform SiO_2_ surface was observed after the removal of the Al layer. After annealing and subsequent etching away of the Al, random localized voids in the SiO_2_ layer, corresponding to areas where Al spiking occurred, were observed on the Al-etched SiO_2_/Si surface with ~1.7% surface coverage by the void regions and with void diameters ranging from 10 to 130 nm, corresponding to an areal density of voids of ~2–8 × 10^8^/cm^2^ for annealing temperatures of 450–600 °C (Supplementary Fig. 3). The density and size distribution of voids do not appear to depend strongly on annealing temperature or duration (Supplementary Fig. 3). AFM imaging of SiO_2_/Si surfaces after annealing and Al etching confirmed that the Al spikes penetrated completely through the SiO_2_ layer but not into the underlying Si, and that the regions between the spikes remained very close to their original thickness, but with slightly increased surface roughness (Supplementary Fig. 6). The corresponding average distance between adjacent voids is considerably smaller than the typical diffusion length of minority carriers in c-Si, enabling the efficient collection of photogenerated holes during photoanode operation. This is a natural consequence of the nature of the Al spiking reaction into thermally grown SiO_2_, and enables the desired spatial distribution of conductive paths to metal catalyst layers to be realized without deliberate lithographic patterning.

### Electrodeposition of Ni catalyst

**Fig. 3:**
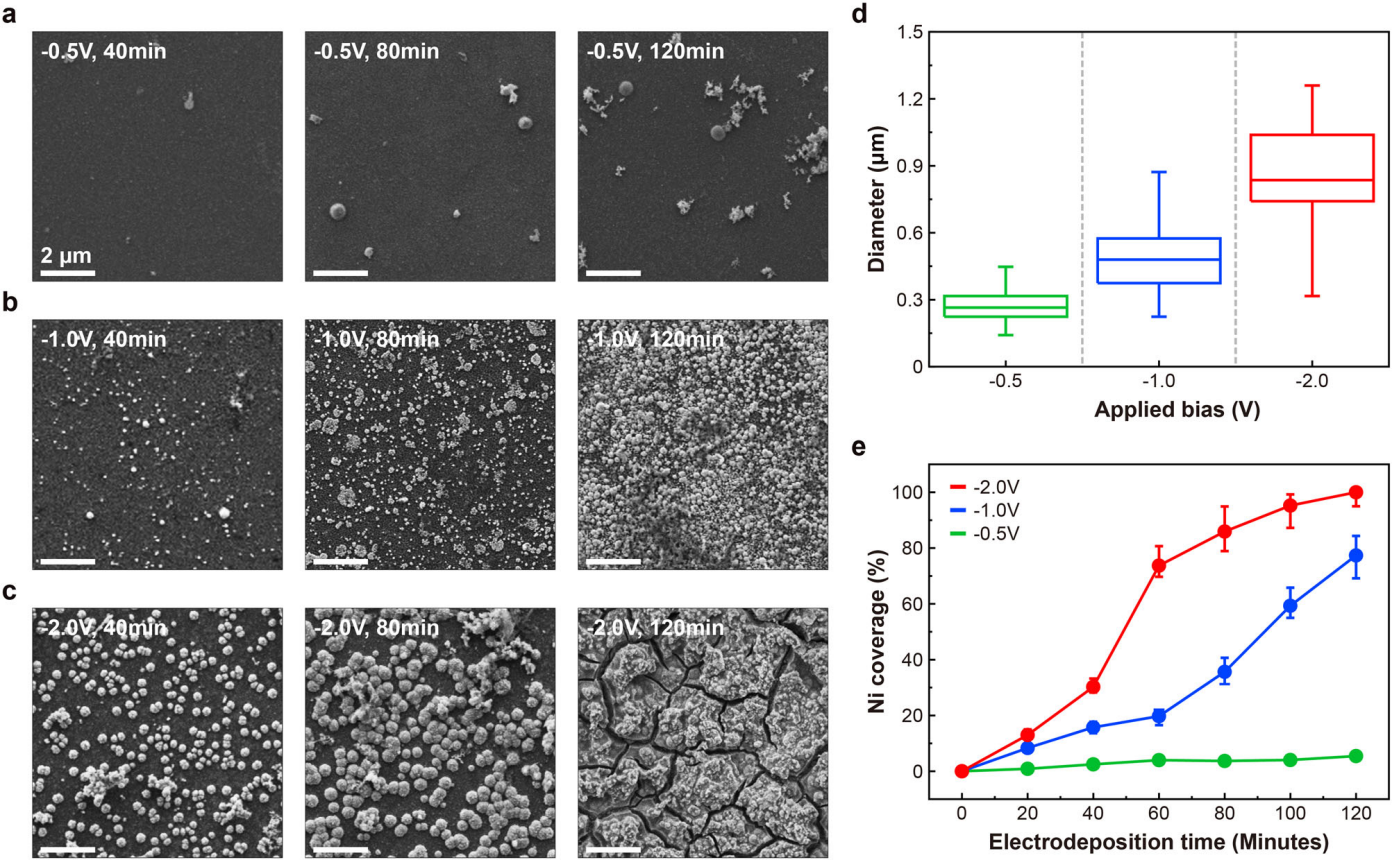
Characterization of Ni electrodeposition. **a**–**c** SEM images of Ni electrodeposited into voids created within a 90 nm SiO_2_ layer on an n-type Si substrate via thin-film reaction with Al, for electrodeposition bias voltages of -0.5 (**a**), −1.0 (**b**), and −2.0 V (**c**) and for the electrodeposition times of 40, 80, and 120 min. All inset scale bars are 2 μm. **d** The size distribution of Ni nano-islands on the SiO_2_/Si surface after 60 min electrodeposition at applied bias voltages of −0.5, −1.0, and −2.0 V. **e** Ni coverage on the SiO_2_/Si surface as a function of electrodeposition time, for electrodeposition bias voltages of −0.5, −1.0, and −2.0 V.

For MIS photoanodes, the metal catalyst layer is essential in enhancing the overall reaction rate, reducing the onset voltage, and increasing the current density. Ni is frequently used as an OER catalyst due to its good electrical conductivity, efficient catalytic effect, and corrosion resistance at high pH^11^. In this study, Ni was incorporated as a catalyst for the photoanode using electrodeposition, following the formation of localized voids in the SiO_2_ protective layer by Al thin-film reaction and etching (Supplementary Fig. 4). Other catalyst materials could readily be employed using this approach. The morphology of Ni deposited on the surface can be controlled by varying the applied bias and deposition time for electrodeposition. When a negative bias is applied to the substrate, Ni deposition begins by filling the voids in the SiO_2_ layer, since the highest electric fields are present at the interface between the aqueous solution and the exposed Si surface. Ni is nucleated at these locations and eventually forms larger islands on the surface. Figure 3a–c shows SEM images of Ni electrodeposited on the SiO_2_/Si surface for different electrodeposition conditions. When low electrodeposition bias (−0.5 V) was applied, Ni nano-islands were observed on the surface and their number and size increased with increasing applied bias and electrodeposition time. Shown in Fig. 3d is a box plot of the diameters of the Ni nano-islands after 80 min of electrodeposition at applied bias voltages of −0.5, −1.0, and −2.0 V. The mean diameter of the Ni nano-islands increased from 0.26 to 0.83 μm as the applied bias increased from −0.5 to −2.0 V. In addition, a broader diameter distribution was observed for the Ni nano-islands deposited at more negative applied bias. Growth and eventually coalescence of the Ni nano-islands occurs with increasing Ni coverage. Figure 3e shows the Ni coverage on the SiO_2_ layer as a function of electrodeposition time. At −0.5 V applied bias, the Ni coverage increased very slowly, not exceeding 5% after 120 min electrodeposition time. When higher bias was applied, clearer increases in Ni coverage were observed with increasing electrodeposition time and, after 120 min, ~78% and ~100% Ni coverage was obtained at −1.0 and −2.0 V, respectively. As shown in Fig. 3c, after 120 min electrodeposition at −2.0 V, the Ni aggregates form a continuous Ni film with a thickness of ~0.3 μm that covers almost the whole surface of the SiO_2_ layer. Cross-sectional SEM imaging and energy-dispersive X-ray spectroscopy (EDS) measurements confirmed that the Ni islands penetrated through the SiO_2_ layer to the underlying Si surface (Supplementary Fig. 5). We refer to photoanodes fabricated in this manner, with Ni metal catalysts penetrating the SiO_2_ protective layer via voids created by the Al spiking and etching processes, as spiked Ni/SiO_2_/Si photoanodes.

### PEC performance for the Ni/SiO_2_/Si photoanodes

**Fig. 4:**
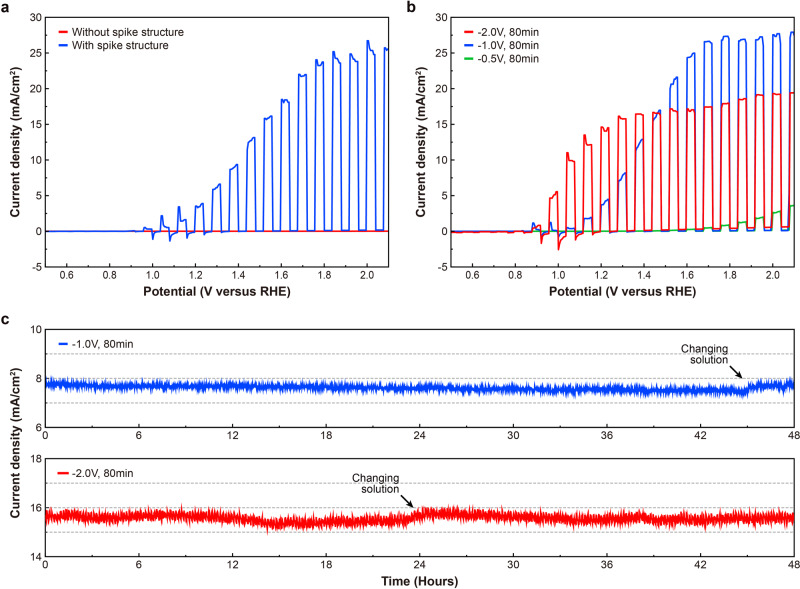
PEC characterization for Ni/90 nm SiO_2_/n-Si photoanodes. **a** LSV curves obtained in 1 M KOH solution with chopped AM1.5G illumination for Ni/90 nm SiO_2_/n-Si photoanodes with (blue) and without (red) Al spiking included in the fabrication process. **b** LSV curves for spiked Ni/90 nm SiO_2_/n-Si photoanodes with Ni electrodeposited for 80 min at −0.5, −1.0, and −2.0 V applied bias. **c** 48 h CA stability tests at −1.3 V versus RHE in 1 M KOH solutions for spiked Ni/90 nm SiO_2_/Si photoanodes with Ni electrodeposited for 80 min at −1.0 and −2.0 V applied bias.

PEC performance was measured for spiked Ni/SiO_2_/Si photoanodes fabricated using various Ni electrodeposition processes in 1 M KOH (pH = 14) aqueous solution with a standard three-electrode system under standard AM1.5G 1sun illumination. Figure 4a shows linear sweep voltammetry (LSV) measurements with chopped illumination for Ni/SiO_2_/Si photoanodes fabricated with and without the Al thin-film reaction process. Both photoanodes were fabricated with the same Ni coverage on the surface. For the spiked structures, the Ni layer was deposited by electrodeposition at −2.0 V applied bias for 60 min yielding ~75% surface coverage with electrodeposited Ni. For the photoanode fabricated without Al spiking, the Ni layer was deposited using e-beam evaporation with the same 75% coverage as the electrodeposited Ni layer using a lithographically defined pattern consisting of 60 μm diameter dots in a square array with 60 μm pitch. As shown in Fig. 4a, the Ni/SiO_2_/Si photoanode without spiking showed very low current density, under 10 μA/cm^2^. This current density is at the noise-limited detection limit of our measurement system, indicating that there is minimal tunneling of the photogenerated charges through the thick SiO_2_ layer. Clear OER activity was observed with the spiked structure, which showed a low onset potential of 1.0 V versus reversible hydrogen electrode (RHE) and a high saturation current density of 25 mA/cm^2^. This result confirms that the spiked structure dramatically improves the performance in the aqueous solution of the Ni/SiO_2_/Si photoanode by providing conductive paths through the SiO_2_ layer. All results reported below are for spiked Ni/SiO_2_/Si photoanode structures fabricating using the Al thin-film reaction process.

Figure 4b shows the PEC performance of spiked Ni/SiO_2_/Si photoanodes for different electrodeposited Ni morphologies on the surface. We prepared three spiked Ni/SiO_2_/Si photoanodes with different electrodeposition recipes. The measured Ni coverages of the photoanodes were 3.9%, 34.4%, and 80.2% for 80 min of Ni electrodeposition at −0.5, −1.0, and −2.0 V applied biases, respectively. The spiked Ni/SiO_2_/Si photoanode with 3.9% Ni coverage showed poor PEC performance due to insufficient Ni coverage on the surface. The highest saturation photocurrent density (27 mA/cm^2^) was observed for 34.4% Ni coverage, achieved by Ni electrodeposition at −1.0 V bias. For the spiked Ni/SiO_2_/Si photoanode with 80.2% Ni coverage, a lower saturation photocurrent density (16 mA/cm^2^) was observed compared to the photoanode with 34.4% of Ni coverage, a reduction we attribute to blocking of incident light by the Ni layer. However, an enhancement in onset potential for OER was observed, from 1.1 to 0.9 V versus RHE, for 80.2% Ni coverage on the surface compared to that for 34.4%. This result indicates that, unsurprisingly, the catalytic effect of Ni was improved with increasing Ni coverage, leading to lower onset potential. However, excessive Ni coverage limits light absorption in the underlying Si absorber, reducing the photocurrent density.

The corrosion resistance of the spiked Ni/SiO_2_/Si photoanodes at high pH was assessed by chronoamperometry (CA). As shown in Fig. 4c, CA tests for spiked Ni/SiO_2_/Si photoanodes with different Ni electrodepositions at −1.0 and −2.0 V were performed in 1 M KOH (pH = 14) aqueous solution at 1.3 V versus RHE for 48 h. Due to the 90 nm thick SiO_2_ layer and the high corrosion resistance of Ni for high pH solutions, both spiked Ni/SiO_2_/Si photoanodes showed excellent stability over the entire 48 h measurement duration (Supplementary Fig. 7). Due to the higher rate of gas evolution for the photoanode with Ni electrodeposition at −2.0 V, more fluctuations in photocurrent density were observed during the stability test. A small decrease in photocurrent density was observed for both photoanodes during the stability test, since some of the produced O_2_ bubbles remain attached to the surface and suppress the OER reaction. After drying the surface using Ar dry gas and changing to a new 1 M KOH aqueous solution, the photocurrent density recovered to the initial values, indicating that there was no chemical corrosion on the surface.

### Numerical analysis of photoanode potential distributions

**Fig. 5:**
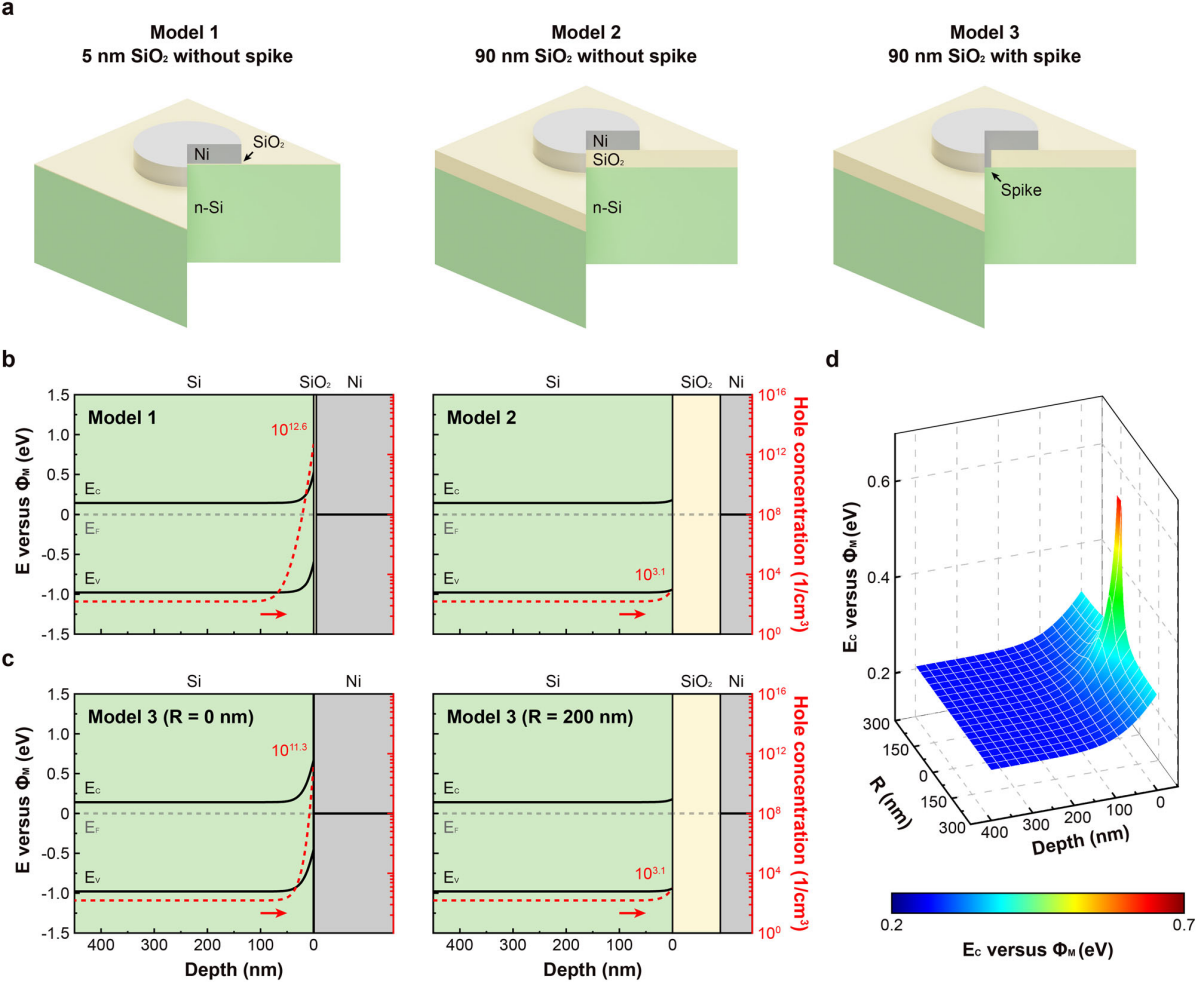
Simulations showing potential distributions for different models. **a** Schematic illustrations of 3D simulation geometries for MIS photoanodes: Ni/5 nm SiO_2_/n-Si without spike for Model 1, Ni/90 nm SiO_2_/n-Si without spike for Model 2, and Ni/90 nm SiO_2_/n-Si with a 60 nm diameter spike for Model 3. **b** Simulated band-edge energy diagrams and hole concentrations at the interface area for Models 1 and 2. **c** Simulated band-edge energy diagrams and hole concentrations for Model 3, at radial distances from the center of the spike, *R*, of 0 and 200 nm. **d** Simulated conduction-band-edge energy (*E*_C_) profile near the spiked area in Model 3.

The effects of localized contacts in the spiked Ni/SiO_2_/Si photoanode were also analyzed computationally using a commercial numerical solver, COMSOL Multiphysics, to help explain the favorable onset potentials observed (Fig. 5 and Supplementary Fig. 8). Three different models were simulated, consisting of different Ni/SiO_2_/Si structures with metal back contacts (Fig. 5a). Models 1 and 2 were designed as typical MIS structures without metal spikes, with SiO_2_ thicknesses of 5 and 90 nm, respectively. Model 3 had a similar structure to Model 2, but also included a cylindrical metal spike with a diameter of 60 nm and a Schottky contact between Ni and Si at the bottom of the spike. Figure 5b, c shows the simulated band structures and hole concentrations of Models 1, 2, and 3. The electrical behavior of Models 1 and 2 is readily explained by the MIS capacitor model^36^. The band structure of a typical MIS contact with a very thin insulating layer is nearly Schottky-like, as shown in Fig. 5b. Due to the high work function of Ni (5.0 eV) and small potential difference across the thin SiO_2_ layer, there is weak surface inversion at the Si surface and a favorable onset potential would be expected. With the increasing thickness of the SiO_2_ layer, even weaker surface inversion is observed with less band bending in the Si layer. Therefore, a lower concentration of holes accumulates at the Si surface for Model 2, which has a thicker SiO_2_ layer, causing decreased photovoltage^36^. As shown in Fig. 5c, two types of interfaces are present in Model 3 depending on radial distance (R) from the spiked area: Si/Ni at *R* = 0 nm and Si/SiO_2_/Ni at *R* = 200 nm. For *R* = 200 nm in Model 3, the simulated band structure is almost same as for Model 2. In the case of *R* = 0 nm, Ni is in direct contact with Si, with an 0.67 eV Schottky barrier, leading to stronger surface inversion and higher local hole concentration at the Si surface. Figure 5d shows the simulated conduction-band edge energy (*E*_C_) profile as a function of the radial distance (*R*) from the center of the spiked area and the depth below Si surface. The Si conduction-band edge energy decreases by ~0.5 eV as *R* increases from 0 to 200 nm, so that higher hole concentrations are present in the Si/SiO_2_/Ni structures primarily near the spiked area. In the MIS photoanode with a localized spike structure, the localized high hole concentration on the Si surface is sufficient to lead to a photovoltage improvement of the photoanode compared to that expected for structures with no spiking.

### Spiked Ni/SiO_2_/Si photoanode with p^+^n-Si

**Fig. 6:**
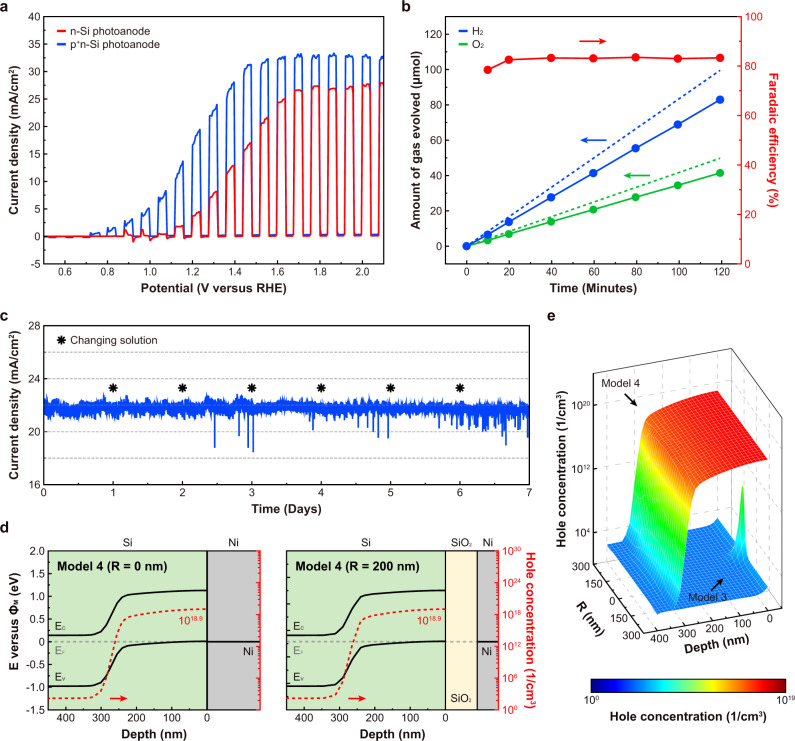
PEC characterization and simulations for the spiked Ni/SiO_2_/p^+^n-Si photoanode. **a** LSV curves obtained in 1 M KOH solution with chopped AM1.5G illumination for spiked Ni/90 nm SiO_2_/n-Si (red) and spiked Ni/90 nm SiO_2_/p^+^n-Si (blue) photoanodes. **b** Ideal (dotted lines) and measured (solid lines and symbols) evolution of H_2_ and O_2_ gases during OER activity at 1.23 V versus RHE for 120 min. The Faradaic efficiency was calculated for O_2_ gas evolution. **c** 7-days CA stability test at −1.3 V versus RHE in 1 M KOH solution. **d** Simulated band diagrams and hole concentrations for Model 4 for *R* = 0 nm and *R* = 200 nm. **e** Simulated hole concentration near the spiked area for Models 3 and 4.

To further improve the photoelectrochemical onset potential, we incorporated a p^+^n-Si junction for the spiked Ni/SiO_2_/Si photoanode. It has been reported that thin p^+^ doping on the n-Si surface improves the OER performance of MIS photoanodes due to the higher hole density at the Si surface^36,37^. The p^+^ doping on the surface was performed by boron diffusion with an expected junction depth of ~300 nm, since a junction depth above 1 μm is expected to increase interface recombination^36^ (Supplementary Fig. 9). Figure 6a shows LSV measurements with chopped illumination for spiked Ni/SiO_2_/Si photoanodes fabricated using p^+^n-Si and n-Si substrates with similar Ni coverage on the surface (~35%). The spiked Ni/SiO_2_/p^+^n-Si photoanode showed enhanced OER performance compared to the spiked Ni/SiO_2_/n-Si photoanode, with a lower onset potential of 0.7 V versus RHE and a higher saturation current density of 32 mA/cm^2^ (Supplementary Fig. 10). In addition, as shown in Fig. 6c, highly stable photocurrent density was observed in a CA measurement for the spiked Ni/SiO_2_/p^+^n-Si photoanode in 1 M KOH aqueous solution at 1.3 V versus RHE, with a constant photocurrent density of 21–22 A/cm^2^ maintained over the entire duration of a 7-days stability test. For the calculation of Faradaic efficiency, the evolved H_2_ and O_2_ gases were measured for the spiked Ni/SiO_2_/p^+^n-Si photoanode at 1.23 V versus RHE. The generation rates of H_2_ and O_2_ gases exhibited the expected stoichiometric ratio (2:1). The calculated Faradaic efficiency started at 72.7% and saturated near 86% at 20 min, indicating good stability for the 120 min measurement (Fig. 6b). It has been reported that saturated faradaic efficiency of ~85% is a typical value for OER due to limiting factors such as the back reaction of the dissolved oxygen^38^, oxidation of Ni catalysts^39^, and facile electrolyte anion oxidation^40^.

The improvement in OER performance for the spiked Ni/SiO_2_/p^+^n-Si photoanode was analyzed by simulating its band structure for a gaussian acceptor doping profile corresponding to a boron diffusion process from the surface with a junction depth of 300 nm, referred to as Model 4 (Supplementary Fig. 11). Figure 6d shows the simulated band structures of Model 4 with *R* = 0 nm and *R* = 200 nm. The spiked (*R* = 0 nm) and non-spiked (*R* = 200 nm) areas in Model 4 showed almost the same band structures, since the p^+^-Si and Ni form an Ohmic contact and band bending in the Si is dominated by the p^+^n junction. The p^+^ doping at the n-Si surface enables a higher hole density to accumulate at the Si surface, as shown in Fig. 6e, leading to improved onset potential compared to the spiked Ni/SiO_2_/n-Si photoanode.

## Conclusion

In summary, we have demonstrated a general method for low-cost, lithography-free, and scalable fabrication of Si-based MIS photoanodes by employing an Al thin-film reaction process combined with Ni catalyst electrodeposition. This approach allows a thick SiO_2_ insulator (90 nm) to be employed, which provides antireflection functionality on Si and high long-term stability in alkaline solutions. Optimization of the Al thin-film reaction process to create a suitable density of openings in the SiO_2_ layer, and of the Ni electrodeposition process to achieve optimal Ni surface coverage, leads to favorable onset potentials and high photocurrent densities. The enhancement in OER performance was analyzed by PEC measurements and numerical simulations using MIS Schottky contact models. In PEC measurements, the optimized Si-based MIS photoanode with a buried p^+^n-Si junction showed low onset potential and high saturation photocurrent density around 0.7 V versus RHE and 32 mA/cm^2^, respectively. Furthermore, the high photocurrent density was maintained for the entirety of a 7-days stability test in a 1 M KOH aqueous solution. These results demonstrate a low-cost, highly scalable approach for the fabrication of efficient and very stable photoanodes that is suitable for large-scale commercial fabrication and readily adaptable to a variety of catalyst, insulator, and semiconductor materials.

## Methods

### Fabrication of Ni/SiO_2_/n-Si photoanodes with localized spike structures

4 inch n-type (100) c-Si wafers (thickness *t* ~ 550 µm and resistivity *ρ* = 0.3–0.5 Ω cm) were used for the fabrication of photoanodes. Each wafer was cleaved into 2 × 2 cm^2^ samples and cleaned with piranha solution (5:1:1 H_2_O:H_2_SO_4_:H_2_O_2_). Cleaned Si substrates were dipped into a 5% HF aqueous solution for 1 min to remove the native oxide. For the back contact, 5 nm Cr and 100 nm Au were deposited on the backside of the Si substrates by e-beam evaporation. High-quality SiO_2_ films were thermally grown on the front side of each Si wafer using an oxidation furnace (MRL 8′ furnace, Sandvik Thermal Process Inc.) at 950 °C in dry O_2_ ambient. In this work, all photoanodes were fabricated with 90 nm SiO_2_ layers. An Al-Si layer with 1% Si content was deposited onto the SiO_2_/Si surface by DC magnetron sputtering (UNIVEX 450B) under 1 × 10^−6^ Torr of base pressure. The use of Si-doped Al suppresses penetration of the Al metallization into the Si layers. For the formation of localized Al spikes through the SiO_2_ layer, Al/SiO_2_/Si substrates were annealed at temperatures of 450–600 °C and durations of up to 24 h in a vacuum chamber. To replace the top Al layer with the Ni catalyst, Al was etched using a 10% H_3_PO_4_ aqueous solution for 6 h. After Al etching, the surface was rinsed by DI water and dried under N_2_ flow. The Ni catalyst was deposited by electrodeposition. The Ni plating solution consisted of a 0.1 M boric acid and 0.1 M NiCl_2_ aqueous solution. During electrodeposition, only the SiO_2_/Si surface from which Al was etched was exposed to the Ni plating solution. The working electrode was connected to the back contact and both the Pt counter electrode and Ag/AgCl (3 M KCl) reference electrode were dipped in the Ni plating solution. The Ni electrodeposition was performed with different applied bias voltages (−0.5, −1.0, and −2.0 V versus Ag/AgCl) with deposition times ranging from 0 to 120 min. The resulting spiked Ni/SiO_2_/Si MIS photoanodes were rinsed in DI water and dried naturally under ambient conditions.

### Fabrication of Ni/SiO_2_/p^+^n-Si photoanodes

p^+^ doping on the surface of 4 inch n-type (100) c-Si wafers was performed by annealing n-Si substrates with a boron solid-state source (BoronPlus^TM^) at 950 °C for 70 min with an N_2_ flow rate of 3.5 L/min. These conditions were designed to yield a Gaussian boron diffusion profile with 300 nm junction depth. The 5nmCr/100nmAu back contact, thermally grown 90 nm SiO_2_ layer, and 100 nm Al layer were fabricated using the same methods as for the fabrication of Ni/SiO_2_/n-Si photoanodes, as described above. After the fabrication of the Al/SiO_2_/p^+^n-Si structure, Al spiking was induced by annealing at 550 °C for 24 h in a vacuum chamber, followed by Al etching. Subsequently, 35% Ni coverage was obtained by electrodeposition at −3.0 V versus Ag/AgCl for 30 min.

### Measurements of resistance change after Al spiking

To evaluate the resistance change after the Al spiking anneal, dual-sided Al/SiO_2_/Si/SiO_2_/Al MIS samples were prepared. First, 20, 30, 40, and 90 nm SiO_2_ layers were grown on both sides of 2 × 2 cm^2^ double-side polished n-type Si substrates using a thermal oxidation furnace at 950 °C in dry O_2_ ambient. Then, 100 nm Al films were deposited on both sides of the resulting SiO_2_/Si/SiO_2_ substrates using DC magnetron sputtering. To induce localized Al spiking, the Al/SiO_2_/Si/SiO_2_/Al samples were annealed at 450, 500, 550, and 650 °C for annealing times ranging from 0 to 24 h. Three Al/SiO_2_/Si/SiO_2_/Al samples were prepared for each annealing recipe for the measurements. Before and after each annealing process, the top and bottom Al layers were connected to anode and cathode, respectively, and linear sweep voltammetry (LSV) was performed for evaluating the resistance between the top and bottom of each sample.

### PEC measurements

All PEC measurements were performed using a CHI 760E electrochemical workstation (CH Instruments, Austin, United States) with a standard three-electrode electrochemical cell consisting of Pt wire as a counter electrode and an Ag/AgCl reference electrode. The alkaline solution (pH = 14) for the OER characterization consisted of a 1 M KOH aqueous solution (semiconductor grade, Sigma-Aldrich, 99.99% trace metal basis). The measured potentials versus Ag/AgCl were converted to potential versus reversible hydrogen electrode (RHE) using the following equation:

1 $${E}_{{\rm{RHE}}}={E}_{{\rm{Ag}}/{\rm{AgCl}}}+0.197{\rm{V}}+0.059\times {\rm{pH}}$$

The LSV and chronoamperometry (CA) measurements were carried out for the photoanodes under 100 mW/cm^2^ light illumination using a Xenon arc lamp (66475, Newport) with an AM1.5G optical filter for characterization of OER efficiency and long-term stability tests. To evaluate the energy conversion of photoanode, the applied bias photon-to-current efficiency (ABPE) was calculated using the following equation^41^:

2 $${\rm{ABPE}}=\frac{{P}_{{\rm{out}}}-{P}_{{\rm{in}}}}{{P}_{{\rm{light}}}}\,=\frac{{J}_{{\rm{ph}}}({V}_{{\rm{redox}}}-{V}_{{\rm{bias}}})}{{P}_{{\rm{light}}}}\,$$

where *V*_redox_ is the redox potential for water splitting, *V*_bias_ refers to the potential difference between the working and counter electrodes, *P*_light_ is the light intensity, and *J*_ph_ is the measured photocurrent density. The H_2_ and O_2_ gas evolution were measured by H_2_ and O_2_ microsensors connected to a picoammeter (Unisense A/S, Denmark), respectively, in 1 M KOH solution at 1.23 V versus RHE under illumination. The faradaic efficiency (FE) of photoanode was calculated using the following equation^41^:

2 $${\rm{FE}}=\frac{{\rm{Measure}}\,{\rm{dgas}}\,{\rm{evloution}}}{{\rm{Theoretical}}\,{\rm{gas}}\,{\rm{evolution}}}=\frac{{\rm{Measured}}\,{{\rm{O}}}_{2}\,{\rm{evolution}}}{\left(\frac{{J}_{{\rm{photo}}}\times A\times T}{e}/4\right)/{N}_{{\rm{A}}}}\times 100 \%$$

where *J*_photo_ is the photocurrent density (A/cm^2^), *A* is the illumination area (cm^2^), *T* is the measurement time (sec), *e* is the charge magnitude of an electron (1.602 × 10^−19^ C), and *N*_A_ is Avogadro’s constant (6.02 × 10^23^ mol^−1^).

### Characterization

The optical reflectance of the Ni/SiO_2_/Si substrate was measured using a single grating monochromator optronics laboratory platform (OL 750). The surface and cross-sectional morphologies of spiked SiO_2_ and electrodeposited Ni samples were characterized using field emission scanning electron microscopy (Zeiss, USA). The elements on the surface were analyzed using energy-dispersive X-ray spectroscopy (Bruker Quantax EDS for SEM). For analyzing the surface morphology, the surface was scanned in tapping mode using an atomic force microscope (AIST-NT Omegascope). To evaluate the Ni electrodeposited surface, the diameters and surface coverages of voids and electrodeposited Ni nano-islands were calculated using the “Image J” platform. The numerical simulations for the photoanodes were performed using the semiconductor module of a commercial numerical finite-element solver (COMSOL Multiphysics).

## Supplementary information

Supplementary Information

## Data Availability

Data supporting the findings of this study are available within the article and its Supplementary Information files. Additional data are available from the authors upon reasonable request.
