# Supplementary material for: Scalable, highly stable Si-based metal-insulator-semiconductor photoanodes for water oxidation fabricated using thin-film reactions and electrodeposition
Source: Nat Commun. 2021 Jun 25;12:3982. doi: 10.1038/s41467-021-24229-y (PMC8233328; doi:10.1038/s41467-021-24229-y)
Supplement: Supplementary file 1 — Supplementary Information [file 41467_2021_24229_MOESM1_ESM.pdf]

Supplementary Information for

Scalable, highly stable Si-based metal-insulator-semiconductor photoanodes for water oxidation fabricated using thin-film reactions and electrodeposition

Soonil Lee[1], Li Ji[1,2], Alex C. De Palma[1], and Edward T. Yu[1]

[1] Microelectronics Research Center, University of Texas, 10100 Burnet Rd, Building 160, Austin, Texas 78758, United States

[2] State Key Laboratory of ASIC and System, School of Microelectronics, Fudan University, Shanghai, 200433, China

Correspondence to: ety@ece.utexas.edu and lji@fudan.edu.cn

Optical property of SiO_2 layer

In this paper, a 90 nm thick SiO_2 layer was used for the Ni/SiO_2/Si photoanode. There are two reasons for choosing 90 nm as a thickness of SiO_2 layer. First, the thick SiO_2 layer (greater than 50 nm) can more effectively protect the Si surface from corrosion in the alkaline solutions.[1] Second, the 90 nm SiO_2 layer also provides anti-reflection functionality in water for the 400 ~ 900 nm wavelength range. As shown in Supplementary Fig. 1a,b, the lower light reflectance (under 30%) was observed in both air and water for the SiO_2/Si substrate with 90 nm SiO_2 layer for the wavelength range from 400 to 900 nm, compared to bare Si substrate. Therefore, 90 nm thick SiO_2 layer allows high stability and efficiency for the MIS photoanode with anti-corrosion and anti-reflection properties.

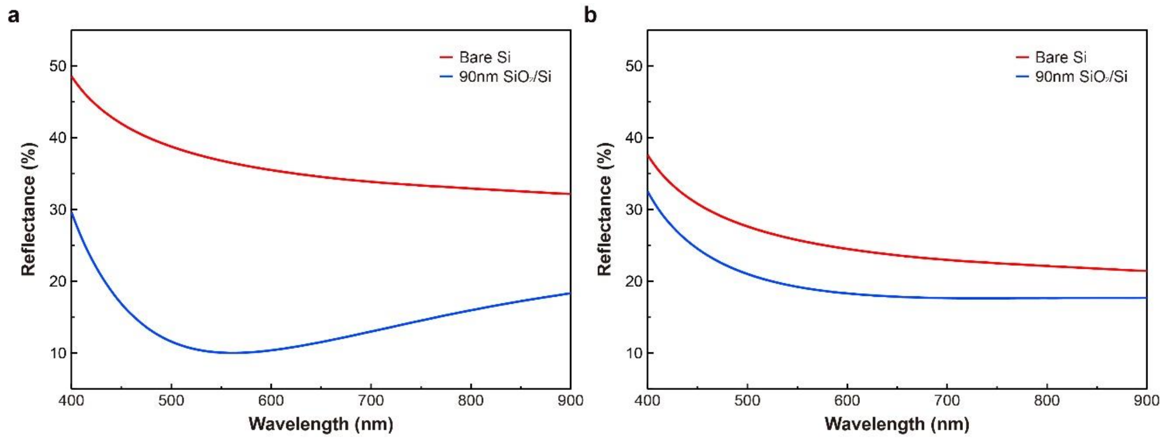

Supplementary Figure 1 Reflectance spectra for the 90nm SiO_2/Si substrate

a,**b**,The calculated reflectance spectra of bare Si and 90nm SiO_2/Si substrates in the air (**a**) and water (**b**) for the different wavelengths of incident light.

Al thin-film reaction for the different SiO_2 layer thicknesses

To investigate the effects of SiO_2 layer thickness on the Al thin-film reaction, the series resistances from top to bottom (top-bottom resistance) of Al/SiO_2/Si/SiO_2/Al samples were measured for SiO_2 layer thicknesses of 20, 30, and 40 nm. As shown in Supplementary Fig. 2, the high resistances were maintained above 90% of their initial values for the first several hours. After the transition points in the green regions, the resistances decreased to below 10% of the initial values for combinations of sufficiently high annealing temperature and long duration. For all SiO_2 layer thicknesses, similar trends of top-bottom resistance changes were observed. Moreover, Fig. 2a shows the similar transition trend for the Al/SiO_2/Si/SiO_2/Al sample with 90 nm SiO_2 layer thickness. These results indicate that the Al thin-film reaction is controlled primarily by annealing temperature and duration, and depends relatively weakly on SiO_2 thickness within the 20-90nm range of thicknesses characterized here.

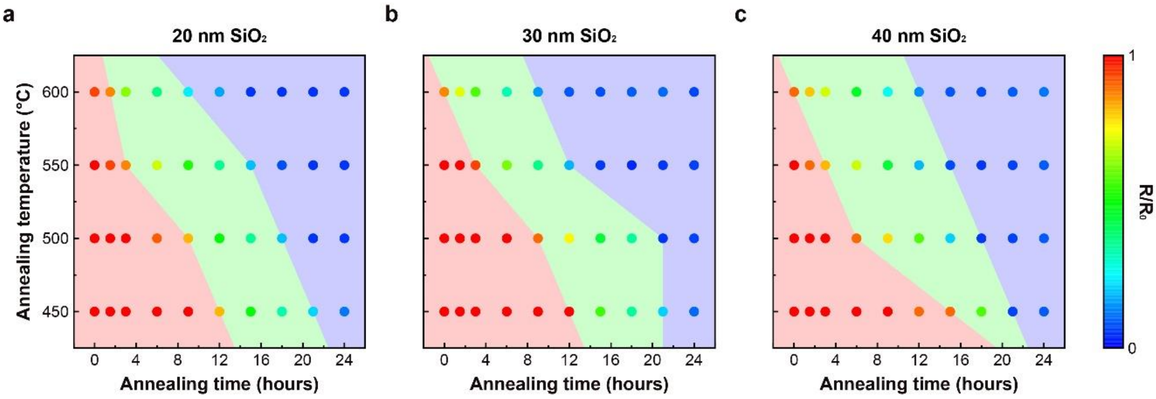

Supplementary Figure 2 Resistance changes after Al spiking

a-c, Electrical resistance of Al/SiO_2/Si/SiO_2/Al structure as function of annealing temperature and duration with SiO_2 layer thickness of 20 nm (**a**), 30 nm (**b**), and 40 nm (**c**). Red, green, and blue regions were above 90%, 10~90%, and below 10% of their initial resistance values.

Distribution of spikes and voids on the SiO_2/Si substrate

After the Al thin-film reaction and etching, the spiked areas are exposed as voids in the SiO_2 layer on the SiO_2/Si substrate. As shown in Supplementary Fig. 3a-e, the random localized voids are observed with similar distributions on the surface for the different annealing temperatures. Supplementary Fig. 3f shows the diameter distribution histogram of voids from the scanning electron microscope (SEM) images in Supplementary Fig. 3b-e. After a 24-hour annealing processes at all temperatures from 450 to 600 °C, similar distributions of void diameters were observed and over 30% of voids have diameters ranging from 50 to 70 nm.

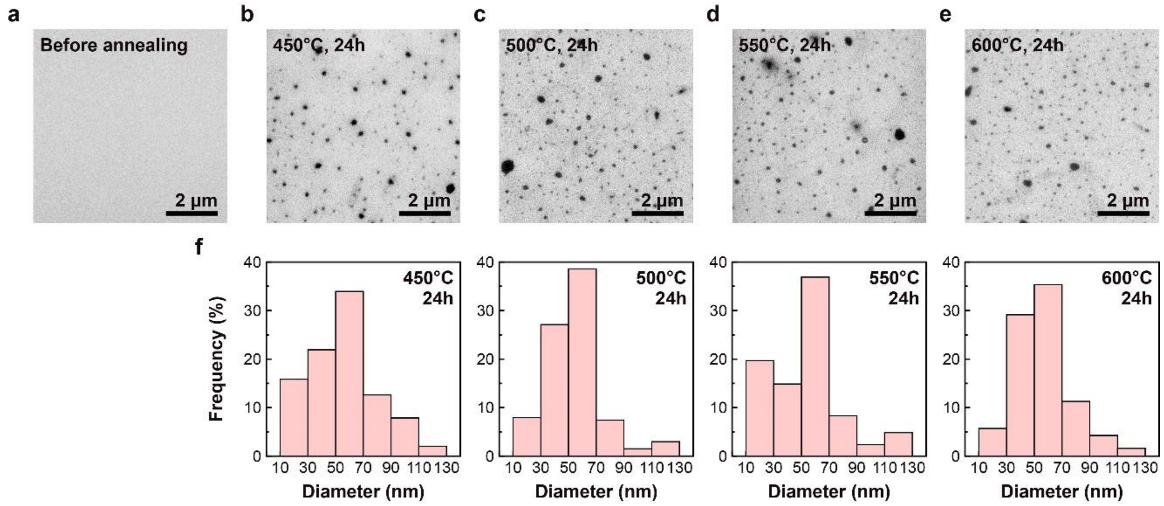

Supplementary Figure 3 SEM images after Al spiking

a-e, Typical plan-view SEM images of SiO_2/Si surfaces after etching of the Al layer before (**a**) and after annealing at 450, (**b**) 500, (**c**) 550, (**d**) and 600 °C (**e**) for 24 hours. **f**, Diameter distribution histograms of voids on the spiked SiO_2/Si surface after each Al thin-film reaction shown in (**b-e**).

Residual Al after etching Al layer

To investigate the potential presence of residual Al and AlO_X after the Al etching process, energy dispersive X-ray spectroscopy (EDX) measurements were performed on the SiO_2/Si substrate after annealing and etching of the Al layer. As shown in Supplementary Fig. 4, No Al peaks were observed on the SiO_2/Si surface, indicating that most or all of the Al and AlO_X were removed by the Al etching process.

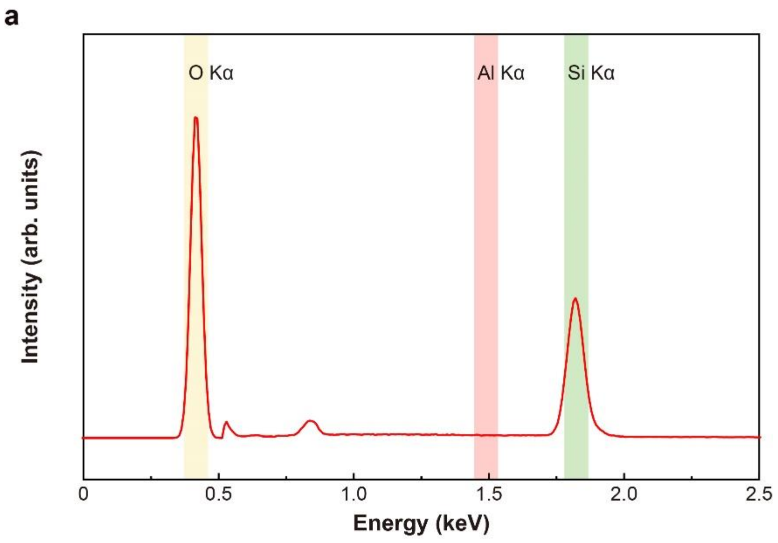

Supplementary Figure 4 EDX spectra after Al etching

EDX spectra of SiO_2/Si surface after Al thin-film reaction and etching.

Cross-sectional SEM imaging of spiked $Ni/SiO_2/Si$ structure

The voids in the SiO_2 layer of the SiO_2/Si substrate created by the Al spiking process are filled and covered by Ni islands after Ni electrodeposition. As shown in Supplementary Fig. 5, cross-sectional SEM imaging and EDX measurements show the direct contact between Ni island and Si substrate through the spike structure of SiO_2 layer. Through the EDX elemental mapping of Ni, O and Si (Supplementary Fig. 5b,c), Ni is seen to be present in the Ni island and spike structure through the SiO_2 layer, and the interface between Ni and Si is observed at the bottom of the spike structure. No penetration of Ni into the Si layer is detected.

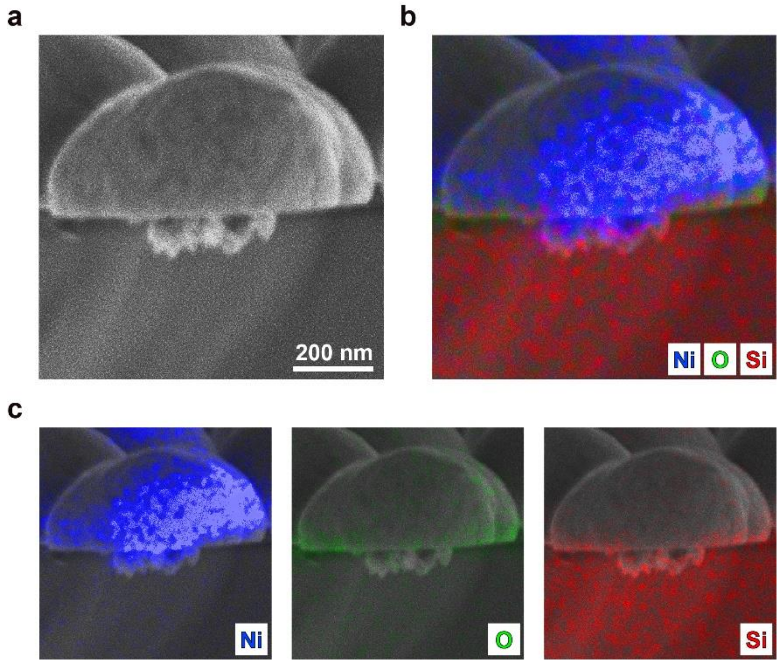

Supplementary Figure 5 Cross-sectional SEM and EDX analysis of $Ni/SiO_2/Si$ substrate

a,**b**, SEM image (**a**) and EDX elemental mapping (**b**) for the $Ni/SiO_2/Si$ substrate. **c**, EDX elemental mapping for the $Ni/SiO_2/Si$ substrate, corresponding to Ni, O, and Si.

Surface morphology of SiO_2 layer

The surface morphologies of bare SiO_2/Si, spiked SiO_2/Si, and spiked Ni/SiO_2/Si substrates characterized using atomic force microscope (AFM) are shown in Supplementary Fig. 6. As shown in Supplementary Fig. 6a, the thermally grown SiO_2 layer has a flat surface. However, after Al thin-film reaction and etching, the spiked SiO_2/Si substrate contains the spiked voids and rough surface. The line profile of Supplementary Fig. 6b shows the voids have depth around 100 nm, confirming that the voids penetrate through the 90 nm SiO_2 layer to reach to the Si layer, and that the full thickness of the SiO_2 layer is maintained. The flat bottom observed for the widest void confirms that penetration into the Si layer does not occur. For smaller voids seen in Supplementary Fig. 6b, the AFM tip may not be able to penetrate to the bottom of the void, reducing the apparent depth of the void. The non-spiked SiO_2 surface area has slightly rougher surface morphology after the Al thin-film reaction compared to the bare SiO_2/Si substrate. However, the original thickness of the SiO_2 layer is still maintained as is evident from the depth and shape of the widest void. As shown in Supplementary Fig. 6c, after Ni electrodeposition, a random distribution of Ni islands with 50~100 nm height is present on the spiked Ni/SiO_2/Si substrate without any observed uncovered spiked voids. This result along with stability measurements shown in Fig. 4c and Fig. 6c indicates that the Ni electrodeposition process completely covers the spiked voids of spiked SiO_2/Si substrate.

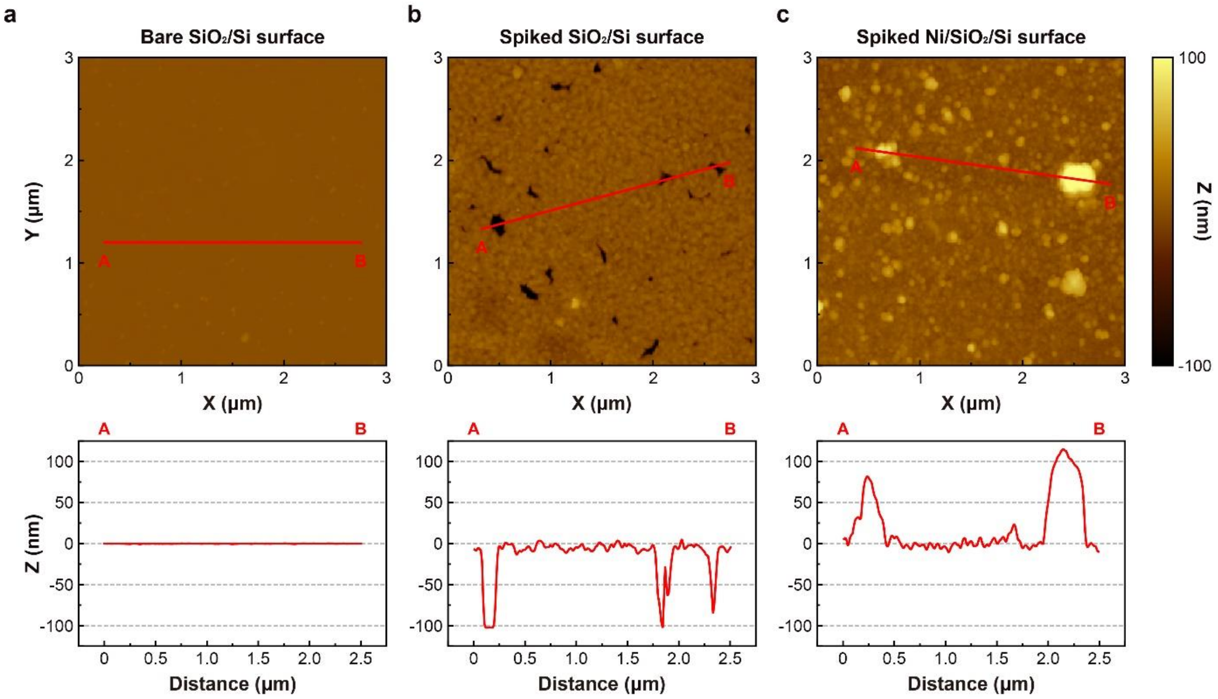

Supplementary Figure 6 AFM images and line profile on the surface

a-c, AFM images and line profiles from A to B of the surfaces of bare SiO_2/Si (**a**), spiked SiO_2/Si (**b**), and spiked Ni/SiO_2/Si (**c**) substrates.

Ni oxidation during stability test

It has been reported that Ni is oxidized to NiOOH during the photoelectrochemical (PEC) reaction in the alkaline solution[2]. To investigate the Ni oxidation during the PEC reaction, SEM and EDX analysis was performed for the Ni/SiO_2/Si photoanode. Supplementary Fig. 7a,b shows the SEM images of the Ni/SiO_2/Si surfaces before and after the 48 hours stability test. The shape of Ni islands changed to particles with rougher surface morphologies. For the further analysis of Ni islands, EDX measurement was performed for the Ni/SiO_2/Si surface before and after the stability test. As shown in Supplementary Fig. 7c,d, higher O Kα peak was observed after the stability test, maintaining Ni Kα peak on the surface. These results indicate that the Ni islands oxidized during the stability test in the alkaline solution, with the CA stability measurements shown in Figs. 4c and 6c indicating that OER functionality is maintained, unaltered, throughout the stability test.

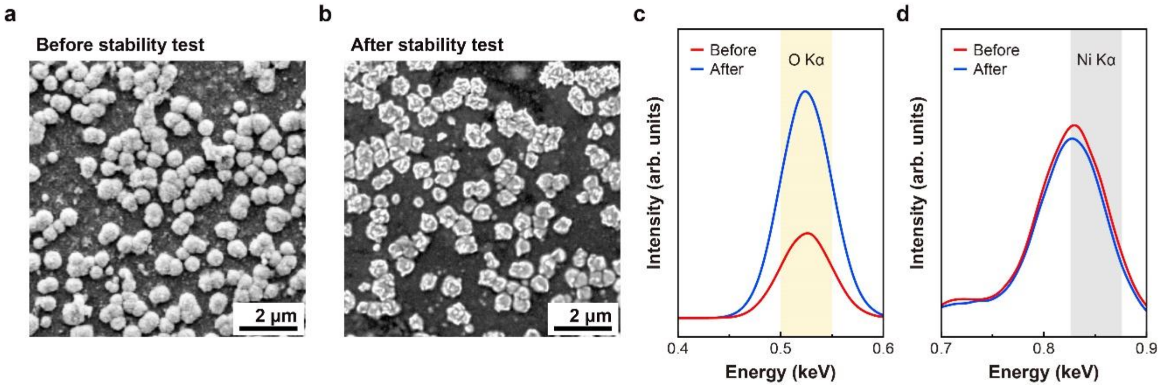

Supplementary Figure 7 SEM and EDX analysis before and after stability test

a,b, Typical plan-view SEM images of Ni/SiO_2/Si surfaces before (**a**) and after (**b**) 48 hours CA stability tests at -1.3 V versus RHE in 1M KOH solution. **c**,**d**, EDX spectra near O Kα (**c**) and Ni Kα (**d**) peaks of Ni/SiO_2/Si surface before and after 48 hours CA stability tests at -1.3 V versus RHE in 1M KOH solution.

Numerical analysis for different Models

Supplementary Fig. 8 shows the COMSOL simulation results obtained at a bias of 1.5 V applied to the bottom contact for Model 1, 2, and 3. In the case of Model 1, higher electric fields are present at the Si/SiO_2 interface compared to Model 2, as expected due to the thinner SiO_2 layer between metal and Si layers and the non-zero electric field within the oxide layer. When lower electric fields are present at the surface, accumulated holes at the interface tunnel through the insulator with lower probability, consistent with a reduction in photocurrent. For Model 3, high electric fields are observed near the spike area. The direct contact between Ni and Si minimizes electrical potential loss on the surface. The low electric fields under the spike area also lower the electric fields of nearby regions under the SiO_2 layer, and this effect diminishes with increasing distance from the spike. Therefore, compared to Model 2, the higher concentration of holes is drifted toward spike area and they can transport to Ni without tunneling, increasing the expected photocurrent.

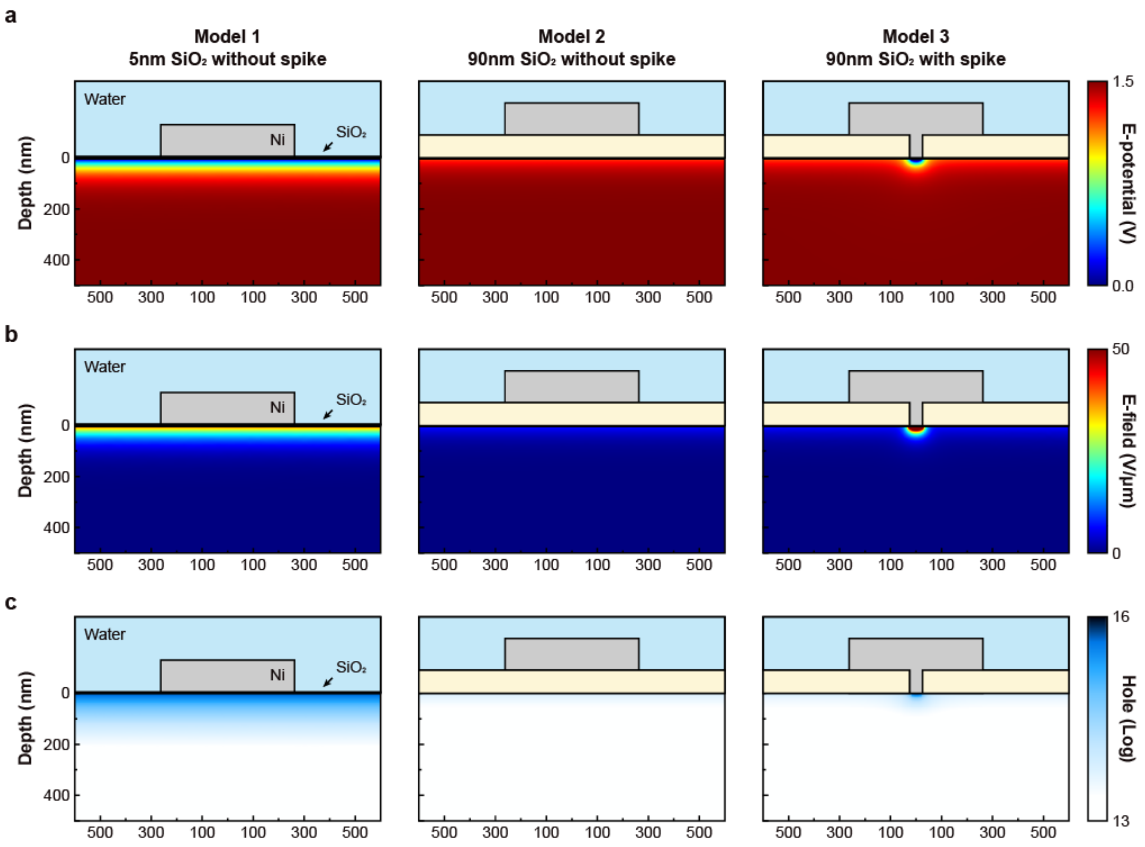

Supplementary Figure 8 COMSOL simulations for different models

a-c, Calculated distribution of electric potential (**a**), electric field (**b**), and hole concentration (**c**) for different photoanode geometries: Ni/5nm SiO_2/n-Si without spike for Model 1, Ni/90nm SiO_2/n-Si without spike for Model 2, and Ni/90nm SiO_2/n-Si with a 60nm diameter spike for Model 3.

pn-junction current-voltage characteristics before and after Ni electrodeposition

To investigate possible damage to the pn-junction of p^+n-Si devices during the Ni electrodeposition process, particularly at large electrodeposition voltages, current-voltage (*I*-*V*) characteristics were measured for the p^+n-Si substrates before and after the Ni electrodeposition. At first, the *I*-*V* curve was measured for the bare p^+n-Si structure before oxidation and electrodeposition, with 300nm Al metallization applied to form an electrical contact. Then for comparison, a Ni/SiO_2/p^+nSi photoanode was fabricated by the usual thermal oxidation and Al etching processes and using Ni electrodeposition at -3.0 V applied bias for 30 min, followed by etching of the Ni and SiO_2 layers to again obtain a bare p^+n-Si substrate. The *I*-*V* curve for the p^+n-Si substrate following these processes, again with 300nm Al metallization for electrical contact, was also measured and compared to that for the original p^+n-Si structure. As shown in Supplementary Fig. 9, both p^+n-Si structures show very similar diode plots, indicating that the Ni electrodeposition at -3.0 V applied bias does not cause any apparent damage to the pn-junction.

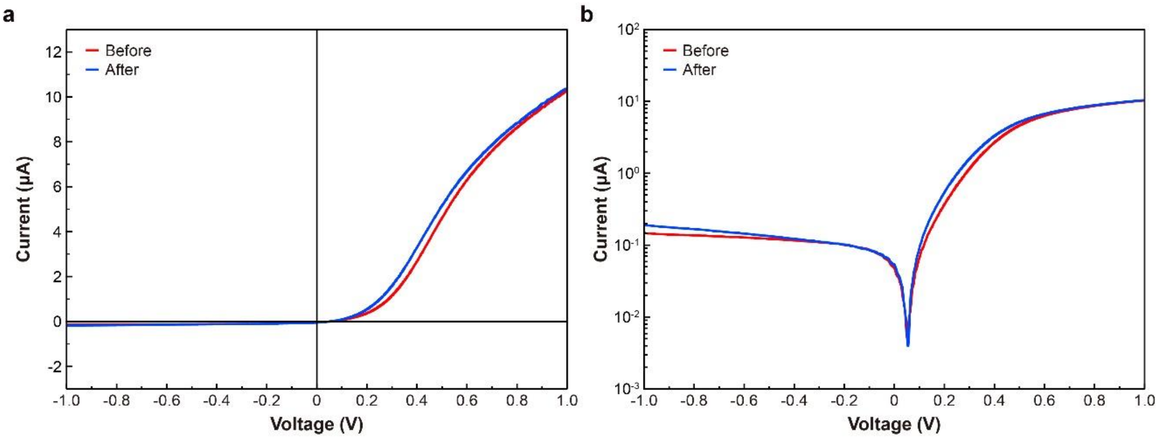

Supplementary Figure 9 *I*-*V* curves of p^+n-Si before and after Ni electrodeposition

a,b, *I*-*V* characteristics of p^+n-Si substrate before and after Ni electrodeposition at -3.0 V applied bias for 30 min with linear (**a**) and log (**b**) scales.

PEC performance of Ni/SiO_2/p^+nSi photoanode

The enhancement in oxygen evolution reaction (OER) performance for the spiked Ni/SiO_2/p^+n-Si photoanode was investigated by analysis with applied-bias photon-to-current efficiency (ABPE) and Tafel plots. As shown in Supplementary Fig. 10a, the spiked Ni/SiO_2/p^+n-Si photoanode achieved an ABPE up to 1.28% at 1.08 V versus RHE which is higher than the spiked Ni/SiO_2/n-Si photoanode (up to 0.17% at 1.15 V versus RHE). Moreover, as shown in Supplementary Fig. 10b, the Tafel slope for spiked Ni/SiO_2/p^+n-Si photoanode was 291 mV/dec, indicating improved hole transport due to the p^+ doping on the n-Si surface, compared to the spiked Ni/SiO_2/n-Si photoanode (225 mV/dec).

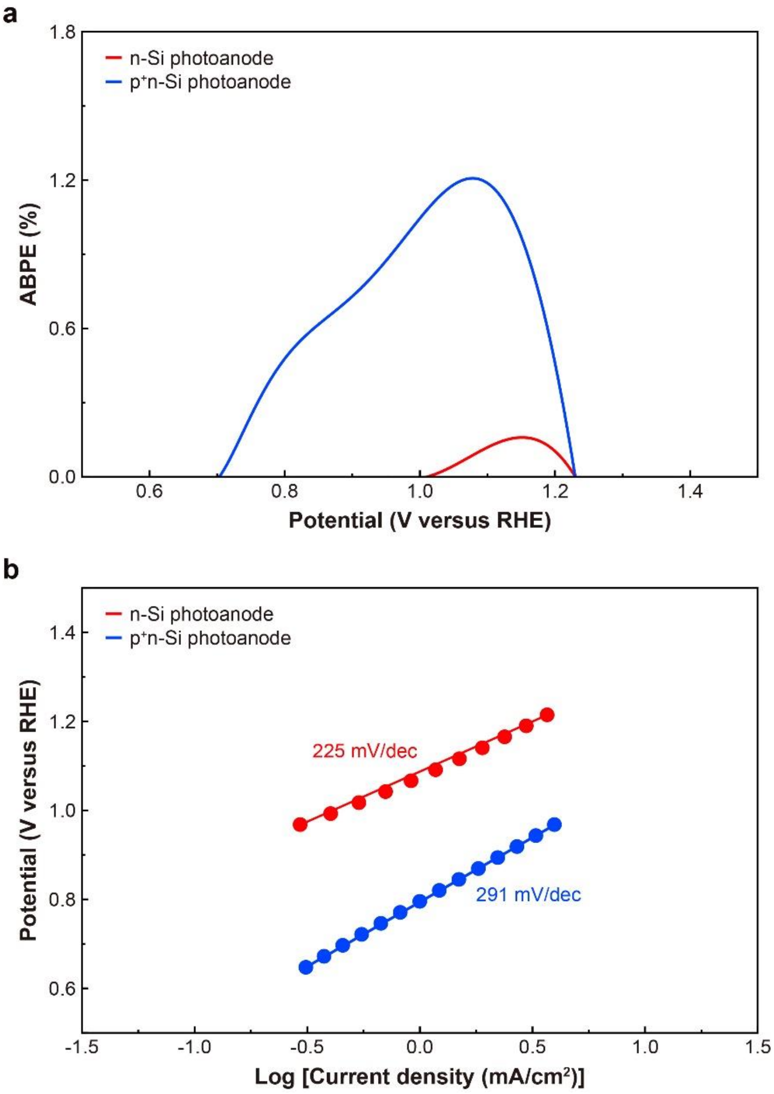

Supplementary Figure 10 PEC performance for the spiked Ni/SiO_2/p$^+$n-Si photoanode

a, ABPE deduced from measured LSV curves as a function of the applied potential versus RHE for the spiked Ni/SiO_2/n-Si and Ni/SiO_2/p$^+$n-Si photoanodes. **b**, Tafel plots corresponding to the LSV curves for the spiked Ni/SiO_2/n-Si and Ni/SiO_2/p$^+$n-Si photoanodes.

Design structure of Model 4

For the numerical analysis of spiked Ni/SiO_2/p^+n-Si photoanode, Model 4 was designed as MIS structure with a p^+n junction. The p^+n Si substrate in Model 4 was determined as a gaussian acceptor doping at the surface of n-Si substrate with a profile in Supplementary Fig. 11. The Model 4 also consists of 90 nm SiO_2 thickness and cylindrical metal spike with a diameter of 60 nm.

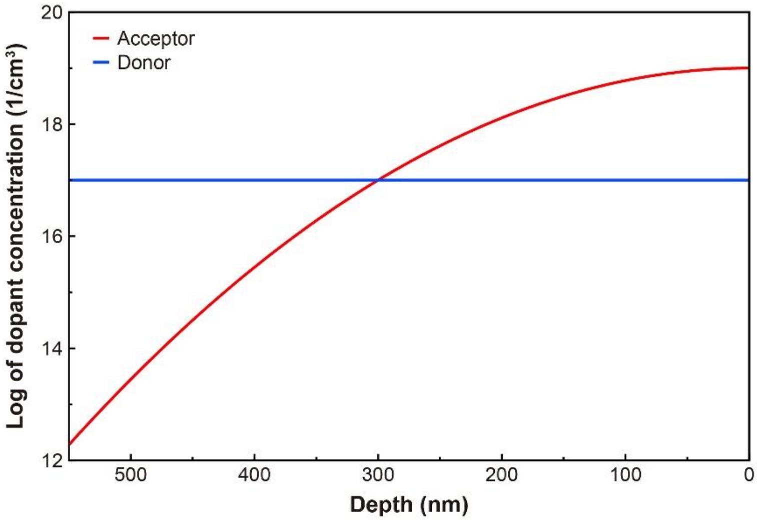

Supplementary Figure 11 Doping profile of the surface of Model 4

Concentrations of acceptors and donors for the p^+n-Si structure used for simulation Model 4.

Supplementary References

1. Zhou, X. *et al.* 570 mV photovoltage, stabilized n-Si/CoOx heterojunction photoanodes fabricated using atomic layer deposition. *Energy & Environmental Science* **9**, 892-897 (2016).

2. Laskowski, F.A.L. *et al.* Nanoscale semiconductor/catalyst interfaces in photoelectrochemistry. *Nature Materials* **19**, 69-76 (2020).
